# SIRT3 deficiency exacerbates fatty liver by attenuating the HIF1α-LIPIN 1 pathway and increasing CD36 through Nrf2

**DOI:** 10.1186/s12964-020-00640-8

**Published:** 2020-09-10

**Authors:** Emma Barroso, Rosalía Rodríguez-Rodríguez, Mohammad Zarei, Javier Pizarro-Degado, Anna Planavila, Xavier Palomer, Francesc Villarroya, Manuel Vázquez-Carrera

**Affiliations:** 1 Department of Pharmacology, Toxicology and Therapeutic Chemistry, Faculty of Pharmacy and Food Sciences, University of Barcelona, Institute of Biomedicine of the University of Barcelona (IBUB), Barcelona, Spain; 2 Spanish Biomedical Research Center in Diabetes and Associated Metabolic Diseases (CIBERDEM)-Instituto de Salud Carlos III, Barcelona, Spain; 3 Research Institute-Hospital Sant Joan de Déu, L’Hospitalet de Llobregat, Spain; 4 Department of Biochemistry and Molecular Biomedicine and IBUB, Faculty of Biology, University of Barcelona, Barcelona, Spain; 5 Spanish Biomedical Research Center in Physiopathology of Obesity and Nutrition (CIBEROBN)-Instituto de Salud Carlos III, Barcelona, Spain; 6 Facultat de Farmàcia i Ciències de l’Alimentació, Unitat de Farmacologia, Farmacognòsia i Terapèutica, Av. Joan XXIII 27-31, E-08028 Barcelona, Spain

**Keywords:** Hepatic steatosis, Sirt3, Nrf2, CD36, VLDLR, NQO1, LIPIN1, HIF-1α, HFD, PPARα

## Abstract

**Background:**

Deficiency of mitochondrial sirtuin 3 (SIRT3), a NAD^+^-dependent protein deacetylase that maintains redox status and lipid homeostasis, contributes to hepatic steatosis. In this study, we investigated additional mechanisms that might play a role in aggravating hepatic steatosis in *Sirt3*-deficient mice fed a high-fat diet (HFD).

**Methods:**

Studies were conducted in wild-type (WT) and Sirt3^−/−^ mice fed a standard diet or a HFD and in *SIRT3*-knockdown human Huh-7 hepatoma cells.

**Results:**

Sirt3^−/−^ mice fed a HFD presented exacerbated hepatic steatosis that was accompanied by decreased expression and DNA-binding activity of peroxisome proliferator-activated receptor (PPAR) α and of several of its target genes involved in fatty acid oxidation, compared to WT mice fed the HFD. Interestingly, *Sirt3* deficiency in liver and its knockdown in Huh-7 cells resulted in upregulation of the nuclear levels of LIPIN1, a PPARα co-activator, and of the protein that controls its levels and localization, hypoxia-inducible factor 1α (HIF-1α). These changes were prevented by lipid exposure through a mechanism that might involve a decrease in succinate levels. Finally, *Sirt3*^−/−^ mice fed the HFD showed increased levels of some proteins involved in lipid uptake, such as CD36 and the VLDL receptor. The upregulation in CD36 was confirmed in Huh-7 cells treated with a SIRT3 inhibitor or transfected with SIRT3 siRNA and incubated with palmitate, an effect that was prevented by the Nrf2 inhibitor ML385.

**Conclusion:**

These findings demonstrate new mechanisms by which Sirt3 deficiency contributes to hepatic steatosis.

**Video abstract**

**Graphical abstract:**

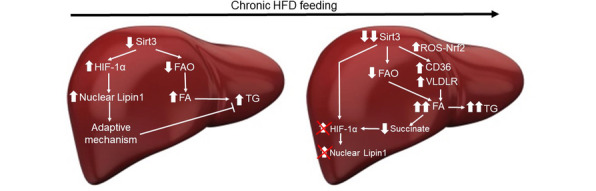

## Background

Mitochondrial sirtuin 3 (SIRT3) is a NAD^+^-dependent protein deacetylase that maintains cellular homeostasis [1]. By deacetylating lysine residues, SIRT3 regulates the activity of many proteins to modulate mitochondrial biogenesis, energy generation and reactive oxygen species (ROS) homeostasis [2]. Thus, previous studies have reported that SIRT3 controls mitochondrial ATP production through its effects on the respiratory chain [1]. Likewise, SIRT3 protects mitochondrial function by modulating ROS generation through several substrates, including superoxide dismutase 2 (SOD2), and the transcription factor Forkhead Box O3A (FOXO3A) [3, 4]. In the liver, SIRT3 acts as a sentinel of redox status, epigenetics, and lipid homeostasis [5]. In addition, mice lacking *Sirt3* fed a high-fat diet (HFD) show enhanced hepatic steatosis compared to wild-type (WT) mice [6], a finding consistent with the hyperacetylation and reduced activity of enzymes involved in mitochondrial fatty acid oxidation [6, 7]. However, it is currently unknown whether Sirt3 prevents hepatic steatosis in mice fed the HFD through additional mechanisms.

Hepatic steatosis is the early stage of non-alcoholic fatty liver disease (NAFLD), the most common liver disorder and one that has reached epidemic proportions worldwide [8]. It can progress to a more severe condition known as non-alcoholic steatohepatitis (NASH), the necroinflammatory form of NAFLD. Several independent factors can stimulate hepatic steatosis, including enhanced de novo lipogenesis, decreased fatty acid oxidation, increased lipolysis, excessive dietary fat consumption, increased fatty acid uptake and changes in the secretion and delivery of lipoprotein particles [9–11]. The fatty acid transporter CD36 (also known as fatty acid translocase) is an important mediator of hepatic fatty acid uptake [12]. In humans, *CD36* expression correlates with triglyceride accumulation in NAFLD [13] and shows a notable increase in the liver of animal models of obesity and type 2 diabetes mellitus [14]. Accordingly, the forced expression of hepatic *Cd36* was accompanied by a marked increase in hepatic fatty acid uptake and triglyceride accumulation [15]. Recently, the very low-density lipoprotein receptor (VLDLR) has also been implicated in the development of hepatic steatosis [16]. This receptor binds apolipoprotein E (apoE) triglyceride-rich lipoproteins such as chylomicrons and VLDL, and intermediate density lipoproteins, thereby causing lipids to enter the cell [17–20]. Given the important role played by CD36 and VLDLR in triglyceride accumulation, identifying the mechanisms that regulate their levels may provide more clues as to how NAFLD develops and progresses.

In this study, we show that *Sirt3* deficiency aggravates HFD-induced hepatic steatosis through a new mechanism that prevents the adaptive increase in the hepatic levels of proteins involved in fatty acid oxidation observed in WT mice fed the HFD, such as LIPIN1. Moreover, *Sirt3*^*−/−*^ mice fed the HFD present increased hepatic levels of proteins involved in lipid uptake through activation of the oxidative stress-induced nuclear factor (erythroid-derived 2)-like 2 (Nrf2). Overall, these findings confirm SIRT3 as a key regulator of NAFLD and shed light on new mechanisms by which the decrease in the levels of this protein may aggravate this disease.

## Methods

### Reagents

Control and Sirt3 siRNA were purchased from Santa Cruz (Dallas, TX). Triglyceride levels were measured using a commercial kit (ref. 1,001,311, SpinReact SA, St. Esteve de Bas, Spain). ML385 (ref. SML1833) and palmitic acid (ref. P5585) were from Sigma (St. Louis, MO) and Albumin, Bovine Serum, fraction V, Fatty Acid-Free (ref. 126,575) was from Merck Millipore (Burlington, MA).

### Mice

Male *Sirt3* knockout mice (B6; 129S5-SIRT3Gt(neo)218Lex) that had been backcrossed into the C57BL/6JOla Hsd line (Harlan Laboratories, Barcelona, Spain) for six generations were obtained from the Mutant Mouse Regional Resource Center (MMRRC). All protocols were approved by University of Barcelona’s Animal Care and Bioethics Committee, in accordance with Law 5/1995, of 21 June passed by Catalan Government, and complied with the EU Directive 2010/63/EU for animal experiments. Male *Sirt3* knockout (*Sirt3*^−/−^) mice (8–10 weeks old) and their wild-type (WT) littermates (*Sirt3*^+/+^) were randomly distributed into two experimental groups (*n* = 6) and fed either standard chow or a HFD (45% fat, mainly from hydrogenated coconut oil, product D08061110, Research Diets Inc., New Brunswick, NJ) for 6 weeks. All animals were killed under anesthetic conditions, and livers were snap-frozen in liquid nitrogen immediately after resection, then stored at − 80 °C.

### Cell culture

Human Huh-7 hepatoma cells (kindly donated by Dr. Mayka Sanchez from the Josep Carreras Leukemia Research Institute) were cultured in DMEM supplemented with 10% fetal bovine serum and 1% penicillin-streptomycin, at 37 °C/5% CO_2_. Lipid-containing media were prepared by conjugation of palmitic acid with fatty acid-free bovine serum albumin, as previously described [21]. siRNA transfections were performed with Lipofectamine 2000 (Life Technologies, Waltham, MA). When indicated, cells were treated with 20 μM of the HIF-1 inhibitor PX478 or 0.5 mM of the cell permeable succinate donor dimethyl fumarate (DMF).

### RNA preparation and quantitative RT-PCR

The relative levels of specific mRNAs were assessed by real-time RT-PCR, as previously described [22]. Primer sequences used for real-time RT-PCR as shown in Table S1.

### Immunoblotting

Isolation of total and nuclear extracts was performed as described elsewhere [21]. Proteins (30 μg) were separated by SDS-PAGE on 10% (w/v) acrylamide separation gels and transferred to Immobilon polyvinylidene difluoride membranes (Merck Millipore). Western blot analysis was performed using antibodies against HIF1α (sc-10,790), LIPIN1 (sc-98,450), Histone H3 (sc-10,809), SREBP1 (sc-365,513), Nrf2 (sc-722), NQO1 (sc-393,736), PPARγ (sc-7273) (Santa Cruz Biotechnology), SIRT3 (#5490), phospho-mTOR Ser2481 (#2974), mTOR (#2972), Keap1 (#4678 s) (Cell Signaling Technology Inc., Danvers, MA), GAPDH (MAB374) (Merck Millipore), CD36 (NB400–144) (Novus Biologicals, Centennial, CO), VLDLR (AF2258) (R&D Systems, Minneapolis, MN). Detection was performed with the Western Lightning™® Plus-ECL chemiluminescence kit (PerkinElmer, Waltham, MA, USA). The size of detected proteins was estimated using protein molecular-mass standards (Bio-Rad, Hercules, CA).

### Reactive oxygen species

The content of reactive oxygen species (ROS) was determined using the Oxiselect™ assay kit (Cell Biolabs Inc., San Diego, CA).

### Succinate levels

Succinate levels were determined using the succinate colorimetric assay kit MAK184 (Sigma). Briefly, 10 mg of liver were homogenized with 100 μL of ice-cold succinate assay buffer and then centrifuged at 10,000 g for 5 min to remove insoluble material. Supernatant was collected and filtered with a 10 kDa spin filter. After adding reaction mix, samples were incubated for 30 min at 37 °C according to manufacturer’s instructions and absorbance was measured at 450 nm.

### Fatty acid uptake assay

Stock solutions of boron-dipyrromethene (BODIPY)-C16 (Invitrogen Life Sciences, Carlsbad, CA) were prepared in DMSO. After treatment with palmitate and/or AAPBO, cells were incubated for 30 min in PBS supplemented with BODIPY-C16 to a final concentration of 100 nM, rinsed with ice-cold PBS and collected in the same buffer. The fluorescence was measured in fresh cells with a microplate fluorescence reader (excitation 490 nm and emission 510 nm).

### Electrophoretic mobility shift assay

The electrophoretic mobility shift assay (EMSA) was performed using double-stranded oligonucleotide for the consensus binding site of the peroxisome proliferator response element (PPRE) (Santa Cruz Biotechnology). Nuclear extracts (NE) were isolated and EMSA was performed as previously reported [21].

### Hematoxylin-eosin staining

We performed hematoxylin-eosin staining as previously reported [21].

### Analysis of intracellular triglyceride content

Total lipids of liver homogenates and cultured cells were extracted according to Bligh and Dyer [22], evaporated, and redissolved in ethanol and triglycerides were determined using a colorimetric kit (Spinreact). Triglycerides were normalized against the total cellular protein content of liver homogenates determined by the Bradford protein assay. In cultured cells, culture media was removed carefully after exposure to different treatments and cells were gently washed with cold PBS, lysed in radioimmunoprecipitation assay (RIPA) buffer and subjected to homogenization. The homogenate was then centrifuged at 15,500 *g* for 15 min at 4 °C to remove the insoluble materials, after which the intracellular levels of triglycerides were measured using a colorimetric kit (Spinreact) and normalized with the cellular content of protein determined by the Bradford protein assay.

### Statistical analyses

Results are expressed as means ± S.D. Significant differences were established by two-way ANOVA using the GraphPad Prism program (GraphPad Software V5.01) (GraphPad Software Inc., San Diego, CA). When significant variations were found by two-way ANOVA, the Tukey-Kramer multiple comparison post-test was performed. Differences were considered significant at *p* < 0.05.

## Results

### Feeding Sirt3^−/−^ mice a HFD exacerbates hepatic steatosis and attenuates the adaptive response involving PPARα

**Fig. 1:**
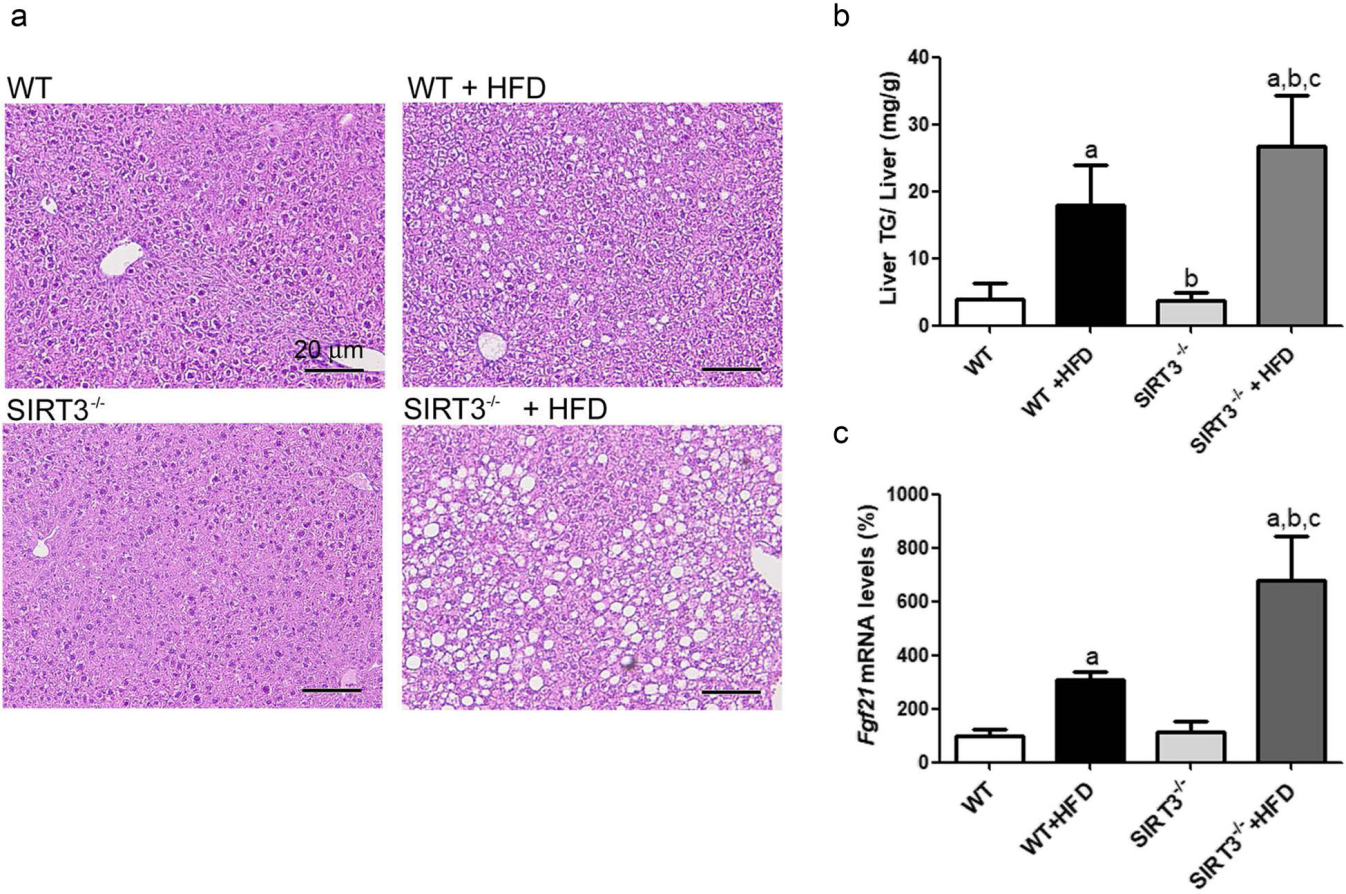
Increased hepatic steatosis *in Sirt3-*deficient mice fed a HFD. **a**, Hematoxylin-eosin staining of livers from male wild-type (WT) and *Sirt3*^*−/−*^ mice fed either a standard chow or the HFD were used (*n* = 6 per group). Scale bar: 20 μm. **b**, liver triglyceride levels. **c**, hepatic *Fgf21* expression. a, *p* < 0.05 vs. WT mice fed a standard chow. b, *p* < 0.05 vs. WT mice fed the HFD. c, *p* < 0.05 vs. *Sirt3*^*−/−*^ mice fed a standard chow

Feeding WT mice the HFD led to hepatic steatosis, as demonstrated by hematoxylin-eosin staining (Fig. 1a) and hepatic triglyceride quantification (Fig. 1b). In line with previous studies [6], HFD-induced hepatic steatosis was aggravated in *Sirt3*^*−/−*^ mice (Figs. 1a-b). Consistent with this finding, the mRNA levels of fibroblast growth factor 21 (*Fgf21*), which reflects liver fat accumulation [23], were higher in *Sirt3*^*−/−*^ mice than in WT when fed the HFD (Fig. 1c).

**Fig. 2:**
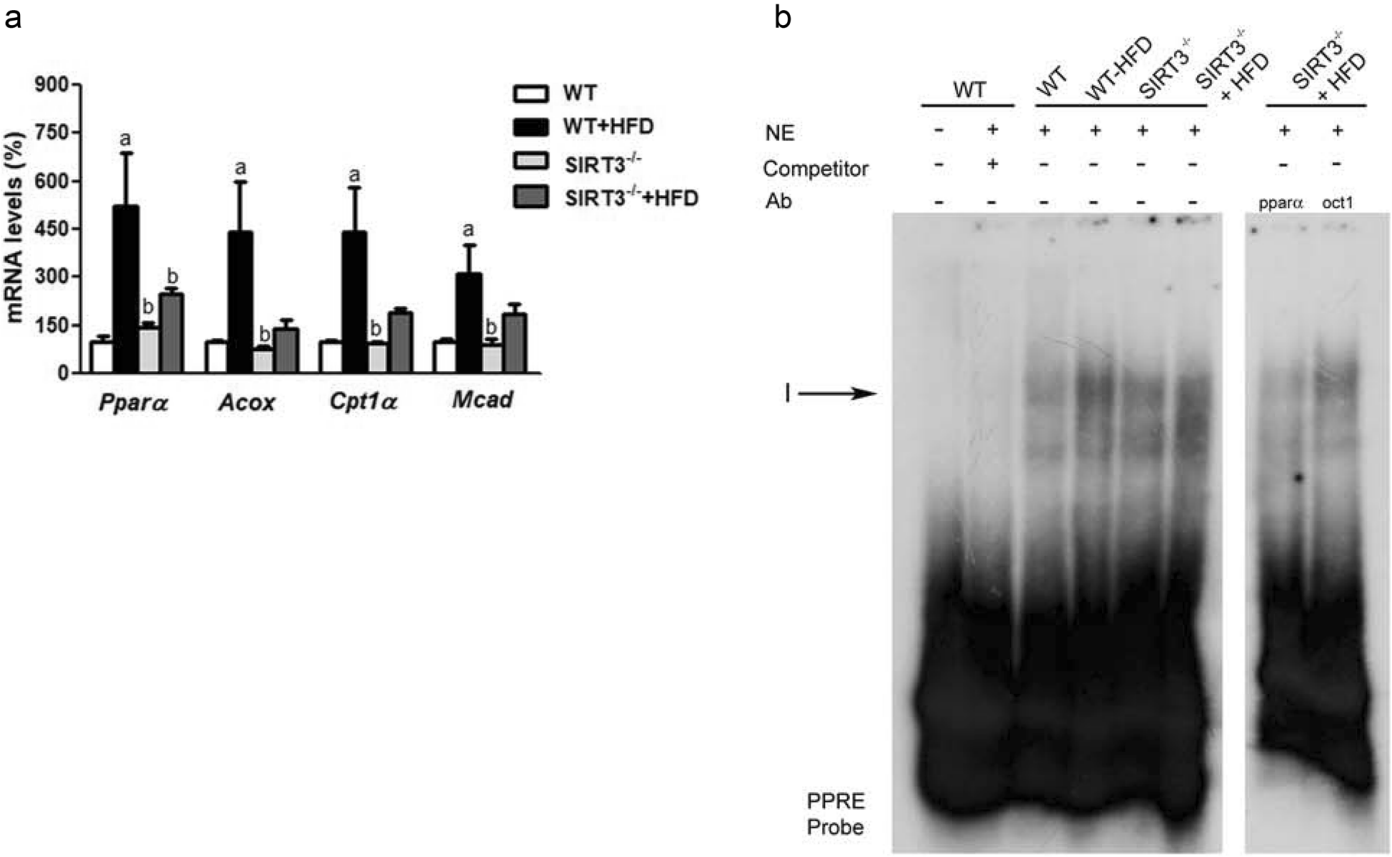
Hepatic dysregulation of the expression of genes involved in fatty acid oxidation in *Sirt3*-deficient mice fed a HFD. **a**, Hepatic mRNA levels of genes involved in fatty acid metabolism of livers from WT and *Sirt3*^*−/−*^ mice fed either a standard chow or a HFD. Data are presented as the mean ± S.D. (*n* = 6 per group). **b**, autoradiograph of EMSA to show PPAR-DNA binding activity was performed with a ^32^P-labeled PPRE nucleotide and crude nuclear protein extract (NE) from livers of WT and Sirt3^−/−^ mice fed either a standard chow or a HFD. a, *p* < 0.05 vs. WT mice fed a standard chow. b, *p* < 0.05 vs. WT mice fed a HFD. c, *p* < 0.05 vs. *Sirt3*^*−/−*^ mice fed a standard chow. Ab, antibody

Dysregulation of fatty acid metabolism in liver provokes hepatic steatosis. Hepatic fatty acids are oxidized to meet energy needs or esterified to synthesize triglycerides, which are either stored within liver parenchymal cells or incorporated into VLDL particles for subsequent secretion. One of the master regulators of fatty acid oxidation in liver is peroxisome proliferator-activated receptor (PPAR)α [24]. WT mice exposed to the HFD exhibited an increased expression of *Pparα* and several of its target genes involved in fatty acid oxidation, such as acyl-CoA oxidase (*Acox*), medium-chain acyl-CoA dehydrogenase (*Mcad*), and carnitine palmitoyltransferase 1*α* (*Cpt-1α*), whereas this adaptive response was partially prevented in the liver of *Sirt3*^*−/−*^ mice fed the HFD (Fig. 2a). In line with these changes, the DNA-binding activity of PPARα assessed by EMSA showed an increase in the nuclear extracts of livers from WT mice fed the HFD, but this increase was blunted in *Sirt3*^*−/−*^ mice (Fig. 2b). The decrease in band I observed in a competition assay with an excess of unlabelled probe demonstrated that this complex was specific. Likewise, the decrease in the complex observed by incubating nuclear extracts with an antibody directed against PPARα indicated that band I contained PPARα, and this decrease was specific for this nuclear receptor since it was not observed when samples were incubated with an unrelated antibody (Organic Cation Transporter 1, Oct1),

### Lipid exposure abolishes the increase in hepatic LIPIN 1 and HIF-1α caused by Sirt3 deficiency: a role for succinate

**Fig. 3:**
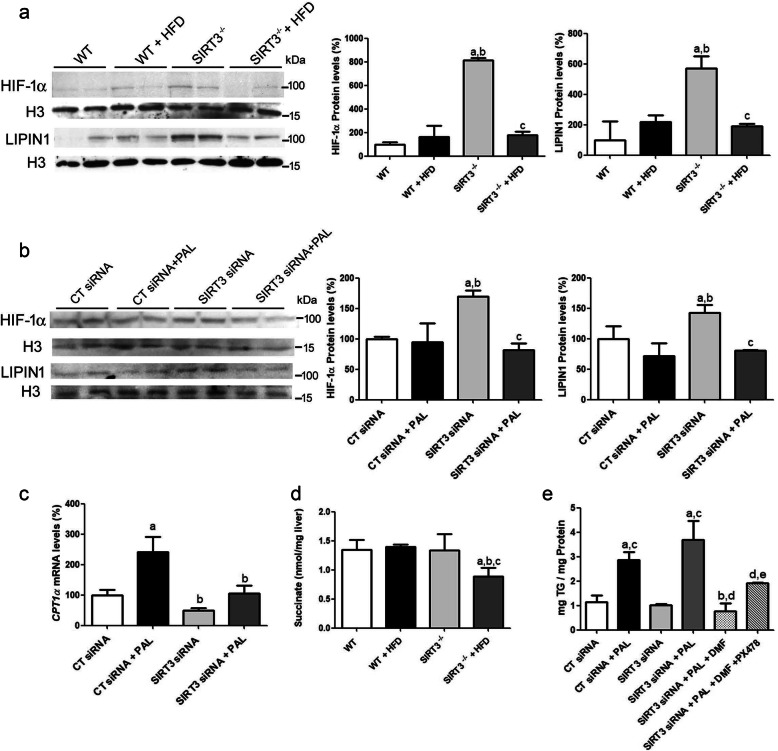
HFD abolishes the increase in hepatic nuclear levels of HIF-1α and LIPIN1 levels observed in *Sirt3*-deficient mice. **a**, hepatic nuclear levels of HIF-1α and LIPIN1 from WT and *Sirt3*^*−/−*^ mice fed either a standard chow or a HFD. Data are presented as the mean ± S.D. (*n* = 6 per group). a, *p* < 0.05 vs. WT mice fed a standard chow. b, *p* < 0.05 vs. WT mice fed a HFD. c, *p* < 0.05 vs. *Sirt3*^*−/−*^ mice fed a standard chow. **b**, nuclear protein levels of HIF-1α and LIPIN1 in Huh-7 cells transfected with control (CT) or *SIRT3* siRNA and incubated with fatty acid free-BSA or BSA-palmitate (0.3 mM) (Pal) for 24 h. **c**, hepatic CPT-1α mRNA levels. a, *p* < 0.05 vs. CT siRNA cells. b, *p* < 0.05 vs. CT siRNA cells incubated with palmitate. c, *p* < 0.05 vs. *SIRT3* siRNA cells. **d**, succinate levels in the liver of WT and *Sirt3*^−/−^ mice fed either a standard chow or a HFD. a, *p* < 0.05 vs. WT mice fed a standard chow. b, *p* < 0.05 vs. WT mice fed a HFD. c, *p* < 0.05 vs. *Sirt3*^*−/−*^ mice fed a standard chow. **e**, triglyceride levels in Huh-7 cells transfected with control (CT) or *SIRT3* siRNA and incubated with fatty acid free-BSA or BSA-palmitate (0.3 mM) (Pal) for 24 h in the presence or absence of DMF and/or PX478. a, *p* < 0.05 vs. CT siRNA cells. b, *p* < 0.05 vs. CT siRNA cells incubated with palmitate. c, *p* < 0.05 vs. SIRT3 siRNA cells. d, *p* < 0.05 vs. SIRT3 siRNA cells incubated with palmitate. e, *p* < 0.05 vs. SIRT3 siRNA cells incubated with palmitate and DMF

Next, we focused on LIPIN 1, since this protein controls whether fatty acids are incorporated into triglycerides or undergo fatty acid oxidation [25]. Thus, in the cytoplasm, LIPIN 1 promotes triglyceride accumulation and phospholipid synthesis by functioning as an Mg^2+^-dependent phosphatidate phosphatase. By contrast, LIPIN 1 in the nucleus acts as a transcriptional co-activator linked to fatty acid oxidation by upregulating PPARα activity, thereby ultimately increasing the expression of its target genes, including *Mcad* and *Cpt-1α* [25]. Previous studies have reported that hypoxia-inducible factor (HIF)-1α regulates LIPIN 1 levels and localization [26, 27]. Moreover, SIRT3 loss represses prolyl hydroxylase domain enzymes, thus leading to increased levels of HIF-1α [28, 29]. Accordingly, *Sirt3*^*−/−*^ mice fed a standard diet presented enhanced nuclear levels of HIF-1α and LIPIN 1, but this was reversed after they were fed the HFD (Fig. 3a). To confirm the effects of *Sirt3* deficiency, human Huh7 hepatoma cells were transfected with siRNA against *SIRT3* in the presence or absence of the saturated fatty acid palmitate. The decline in SIRT3 protein levels caused by siRNA transfection targeting this gene compared to cells transfected with siRNA control (Supplementary Figure 1A and B) was accompanied by increased HIF-1α and LIPIN 1 nuclear protein levels, whereas these changes were prevented in cells incubated with palmitate (Fig. 3b). Moreover, palmitate exposure significantly increased the expression of *CPT-1α*, but this fatty acid-induced increase was blunted in cells transfected with siRNA against *SIRT3* (Fig. 3c). Overall, these findings indicate that the combination of *Sirt3* deficiency and the presence of lipids attenuates the increase in HIF-1α, and therefore in LIPIN 1 levels and the expression of genes involved in fatty acid oxidation, thereby eventually favoring triglyceride accumulation. However, the question that arises is why lipid excess prevents the increase in the levels of HIF-1α caused by *Sirt3* deficiency. Of note, succinate promotes HIF-1α protein stabilization via inhibition of the HIF hydroxylase enzymes and an excess of fatty acids has been reported to reduce succinate, which in turn attenuates HIF-1α levels [30]. To assess this possibility, we examined succinate levels. Only *Sirt3*^*−/−*^ mice fed the HFD presented a decrease in liver succinate levels (Fig. 3d). These data suggest that the robust increase in liver lipids observed in *Sirt3*^*−/−*^ mice fed the HFD reduces succinate concentrations, thereby leading to decreased HIF-1α levels and blunting the adaptation to *Sirt3* deficiency. To clearly confirm the involvement of succinate and HIF-1α in the regulation of triglyceride accumulation, Huh7 cells were exposed to the cell permeable succinate donor DMF and the HIF-1α inhibitor PX478 and triglyceride levels were assessed (Fig. 3e). Incubation of cells with palmitate led to a significant increase in triglyceride accumulation and this was exacerbated in cells transfected with siRNA against *SIRT3*, although differences did not reach statistical significance. Interestingly, the increase in triglyceride levels was completely blunted when cells were incubated with DMF, which is consistent with a role for succinate preventing triglyceride accumulation. However, the effect of DMF was attenuated in cells incubated with DMF and the HIF-1α inhibitor PX478, indicating that the activity of this transcription factor is required to prevent triglyceride accumulation. Thus, in vitro findings confirm that an increase in succinate reverses triglyceride accumulation in Sirt3-deficient cells exposed to palmitate and that this effect of succinate requires the activity of HIF-1α.

### Increased lipogenesis is not involved in increased hepatic steatosis in *Sirt3*^*−/−*^ mice fed the HFD

**Fig. 4:**
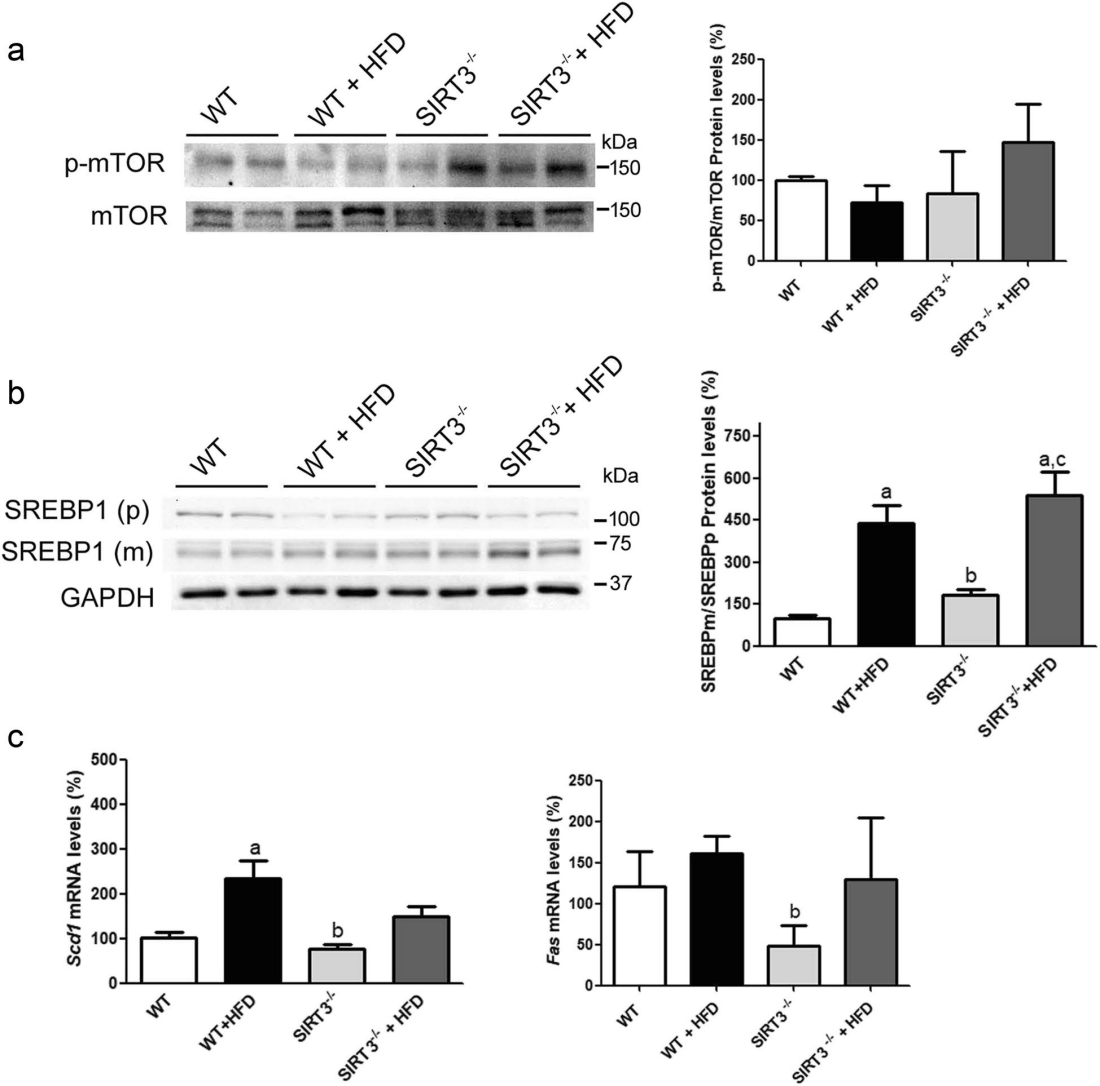
Lipogenesis is not increased in *Sirt3-*deficient mice fed a HFD. **a**, protein levels of total and phospho-mTOR in liver from wild-type (WT) and *Sirt3*^*−/−*^ mice fed either a standard chow or a HFD. **b**, protein levels of precursor (p) and mature (m) forms of SREBP1. **c**, hepatic *Scd1* and *Fas* mRNA levels. Data are presented as the mean ± S.D. (*n* = 6 per group). a, *p* < 0.05 vs. WT mice fed a standard chow. b, *p* < 0.05 vs. WT mice fed a HFD. c, *p* < 0.05 vs. *Sirt3*^*−/−*^ mice fed a standard chow

Next, we examined whether increased lipogenesis was also involved in the exacerbation of hepatic steatosis in *Sirt3*^*−/−*^ mice fed the HFD. Mammalian target of rapamycin (mTOR) complex 1 (mTORC1) regulates sterol regulatory element-binding protein (SREBP), the master regulator of fatty acid biosynthetic gene expression [31], by controlling the nuclear entry of LIPIN 1. Once in the nucleus, LIPIN 1 promotes the downregulation of nuclear SREBP1c abundance. Additionally, mTORC1 is also necessary for activating the processing of SREBP1c to its mature form [32]. Since it has recently been reported that a loss of SIRT3 hyperactivates mTORC1 [33], we assessed whether this pathway was activated in our conditions. Only *Sirt3*^*−/−*^ mice fed the HFD presented an increase in phospho-mTOR, although the differences did not reach statistical significance (Fig. 4a). When we examined the precursor and mature forms of SREBP1, we observed no significant increase in the mature form of this transcription factor in *Sirt3*^*−/−*^ mice fed a HFD compared to WT mice fed the same diet (Fig. 4b). This would suggest that increased fatty acid synthesis was not a key mechanism in the exacerbation of steatosis in *Sirt3*^*−/−*^ mice fed the HFD. Accordingly, the expression levels of two genes involved in lipogenesis, stearoyl-CoA desaturase 1 (*Scd1*) and fatty acid synthase (*Fas*), did not increase significantly in the liver of *Sirt3*^*−/−*^ mice fed the HFD (Fig. 4c).

### Lipid exposure in the presence of Sirt3 deficiency upregulates the levels of proteins involved in lipid uptake through Nrf2

**Fig. 5:**
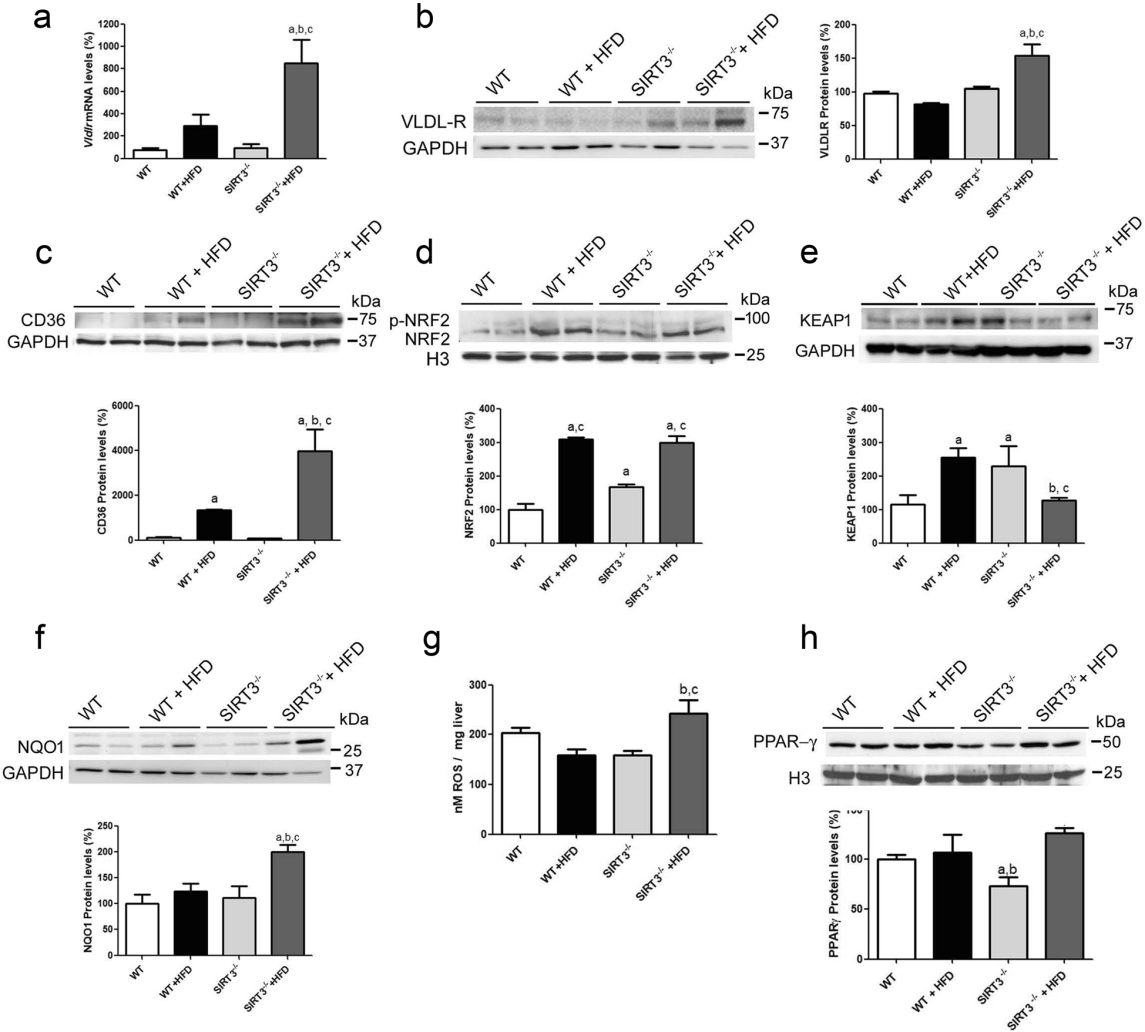
Enhanced hepatic VLDLR and CD36 levels in *Sirt3*-deficient mice fed a HFD. mRNA abundance (**a**) and protein levels (**b**) of VLDLR in livers of WT and Sirt3^−/−^ mice fed either a standard chow or a HFD. CD36 (**c**), total and phospho-Nrf2 (**d**), Keap1 (**e**) and NQO1 (**f**) protein levels. **g**, ROS levels. **h**, PPARγ protein levels. Data are presented as the mean ± S.D. (*n* = 6 per group). a, *p* < 0.05 vs. WT mice fed a standard chow. b, *p* < 0.05 vs. WT mice fed a HFD. c, *p* < 0.05 vs. *Sirt3*^*−/−*^ mice fed a standard chow

**Fig. 6:**
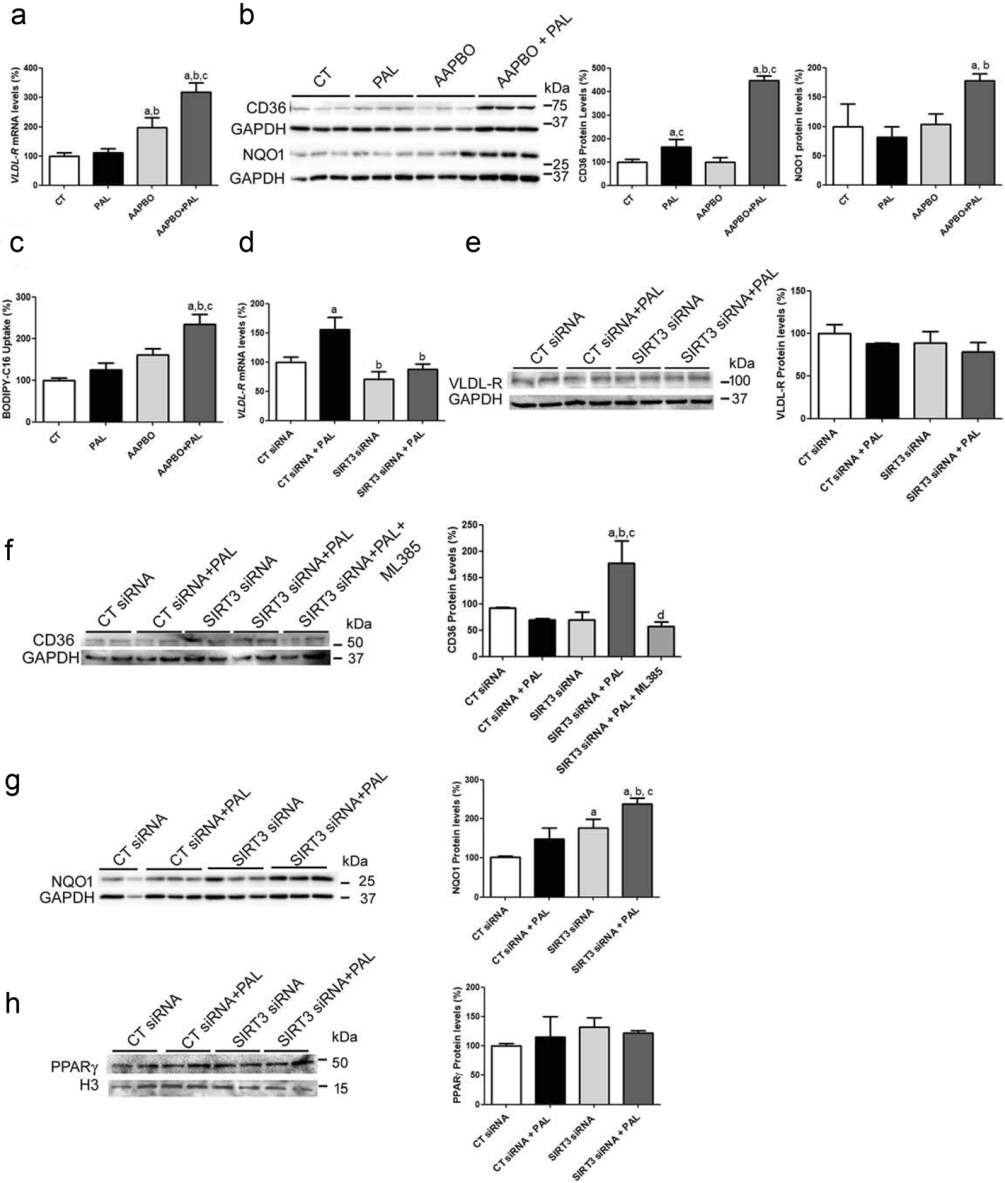
The increase in CD36 levels caused by lipids in *Sirt3*-deficient hepatocytes is mediated by Nrf2. VLDLR mRNA abundance (**a**) and protein levels of VLDLR and NQO1, an Nrf-2-target gene, (**b**) were assessed in Huh-7 cells incubated with fatty acid free-BSA or BSA-palmitate (0.3 mM) and exposed to either vehicle or the Sirt3 inhibitor AAPBO (100 μM) for 16 h. a, *p* < 0.05 vs. CT. b, *p* < 0.05 vs. CT cells incubated with palmitate. c, *p* < 0.05 vs. CT cells treated with AAPBO. **c**, fatty acid uptake in Huh-7 cells incubated with fatty acid free-BSA or BSA-palmitate (0.3 mM) and exposed to either vehicle or the Sirt3 inhibitor AAPBO (100 μM) for 16 h was measured by the uptake of BODIPY-C16. a, *p* < 0.05 vs. CT. b, *p* < 0.05 vs. CT cells incubated with palmitate. c, *p* < 0.05 vs. CT cells treated with AAPBO. mRNA abundance (**d**) and protein levels of VLDLR (**e**) in Huh-7 cells transfected with control (CT) or *SIRT3* siRNA and incubated in the presence or absence 0.3 mM palmitate (Pal) for 24 h. Protein levels of CD36 (**f**), NQO1 (**g**) and PPARγ (**h**) in Huh-7 cells transfected with control (CT) or *SIRT3* siRNA and incubated in the presence or absence 0.3 mM palmitate (Pal) or the Nrf2 inhibitor ML385 (10 μM) for 24 h. a, *p* < 0.05 vs. CT siRNA cells. b, *p* < 0.05 vs. CT siRNA cells incubated with palmitate. c, *p* < 0.05 vs. *SIRT3* siRNA cells. d, *p* < 0.05 vs. CT siRNA cells incubated with palmitate and ML385

We also determined whether the aggravation of hepatic steatosis in *Sirt3*^*−/−*^ mice fed the HFD involved changes in the levels of proteins that favor lipid uptake. First, we checked the VLDLR levels. The expression and protein levels of this receptor increased significantly only in the liver of *Sirt3*^*−/−*^ mice fed the HFD (Fig. 5a and b). With respect to CD36 protein levels, we observed a significant increase in WT mice fed the HFD diet, but this increase was exacerbated in *Sirt3*^*−/−*^ mice fed the same diet (Fig. 5c). Since the expression of both *Vldlr* [34] and *Cd36* [35, 36] is regulated by Nrf2, we examined the levels of this transcription factor and its target genes. Exposure to the HFD caused a similar increase in the nuclear protein levels of Nrf2 in WT and *Sirt3*^*−/−*^ mice (Fig. 5d), whereas the levels of the Nrf2 repressor protein Keap1 (Kelch-like ECH-associated protein 1) were only increased in WT mice fed the HFD diet and in *Sirt3*^*−/−*^ mice fed the same diet (Fig. 5e). However, it has been reported that Nrf2 shows a transient increase [37], and assessing the expression of its target genes is a more effective way of checking its activity. In fact, the protein levels of the Nrf2-target gene NAD(P)H:quinone oxidoreductase (NQO1) were only significantly higher in *Sirt3*^*−/−*^ mice fed the HFD (Fig. 5f), thus suggesting that the activity of Nrf2 was higher in this treatment group. The higher activity of the redox-sensitive transcription factor Nrf2 in the livers of *Sirt3*^*−/−*^ mice fed the HFD was consistent with the increased levels of ROS in this group of animals (Fig. 5g). In addition, CD36 has been shown to be a direct target of PPARγ [36] and the protein levels of this PPAR isotype were reduced in the livers of *Sirt3*^*−/−*^ mice fed a standard diet. However, when these mice were fed the HFD, the PPARγ levels were restored (Fig. 5h). Exposure of Huh7 hepatoma cells to the Sirt3 inhibitor 5-amino-2-(4-aminophenyl) benzoxazole (AAPBO, 100 μM) [38, 39] significantly increased *VLDLR* mRNA levels, and this situation was exacerbated when cells were exposed to palmitate (Fig. 6a). Similarly, the combination of the Sirt3 inhibitor and palmitate increased the protein levels of CD36 and NQO1 (Fig. 6b). In agreement with the changes in CD36 protein levels, cellular fatty acid uptake determined with the BODIPY-labeled C16 fatty acid analog showed an increase in BODIPY-C16 uptake in cells exposed to palmitate and the Sirt3 inhibitor AAPBO (Fig. 6c). In contrast to the effects of the Sirt3 inhibitor, *Sirt3* knockdown did not affect VLDLR mRNA or protein levels (Fig. 6d and e), thus suggesting that the remaining levels of SIRT3 following knockdown can prevent the increase in VLDLR abundance. Finally, *Sirt3* knockdown in the presence of palmitate caused a remarkable increase in CD36 protein levels that was prevented by the Nrf2 inhibitor ML385 [40] (Fig. 6f), thus indicating that this increase might be the result of an increase in Nrf2 activity. Accordingly, the protein levels of NQO1 were upregulated (Fig. 6g), whereas no changes were observed in the PPARγ protein levels (Fig. 6h).

## Discussion

Previous studies have reported that *Sirt3-*deficient mice show accelerated steatosis compared to WT mice because of hyperacetylation and the subsequent reduced activity of mitochondrial metabolic enzymes such as long-chain acyl-CoA dehydrogenase (LCAD) [6]. Here, we present evidence that *Sirt3* deficiency in the context of an excess of lipids exacerbates hepatic steatosis through different new mechanisms, thus affecting the expression of transcriptional co-activators involved in fatty acid oxidation and of genes involved in lipid uptake. In our study, WT and *Sirt3-*deficient mice were fed either a standard diet or the HFD. Under these conditions, WT mice fed the HFD presented an increase in the expression of hepatic genes involved in fatty acid oxidation (*Pparα* and its target genes), probably as an initial adaptive mechanism to overcome the higher lipid supply. This adaptive mechanism involving PPARα and its target genes was abrogated in the liver of *Sirt3*-deficient mice fed the HFD. The findings of this study show that the increase in the DNA-binding activity of PPARα and the expression of its target genes are abolished in the liver of *Sirt3-*deficient mice fed the HFD. This indicates that the absence of SIRT3 accelerates fatty acid accumulation and liver hepatic steatosis. In fact, the expression of *Pparα* and its target genes is upregulated in wild-type mice fed the HFD for 6 weeks. In contrast, the increase in the expression of *Pparα* and its target genes observed in wild-type mice fed the HFD was not observed in *Sirt3-*deficient mice fed the HFD, suggesting that the deficiency of this sirtuin accelerates the accumulation of lipids. Further studies are needed to clarify what is the contribution in the decrease of the expression of these genes involved in fatty acid oxidation to the increased hepatic triglyceride accumulation. Overall, the findings of this study suggest that *Sirt3* deficiency in the context of an excess of lipids accelerates the accumulation of lipids and the subsequent processes that prevent the increase in the expression of genes involved in fatty acid oxidation.

The lack of induction in PPARα in the liver of *Sirt3-*deficient mice fed the HFD might involve a decrease in the nuclear levels of LIPIN 1, a PPARα transcriptional co-activator [25]. LIPIN 1 levels and localization are regulated by HIF-1α [26, 27], a transcription factor, which in turn is upregulated by the loss of *Sirt3* [28, 29]. The lack of increase in nuclear LIPIN 1 in the liver of *Sirt3-*deficient mice fed the HFD was accompanied by a decrease in HIF-1α compared to *Sirt3-*deficient mice fed a standard diet. Knockdown of *SIRT3* in human hepatoma cells confirmed the upregulation in both HIF-1α and LIPIN 1 protein levels, whereas this increase was blunted when cells were exposed to palmitate. Therefore, *Sirt3* deficiency provokes an increase in HIF-1α levels that might trigger an adaptive mechanism, which promotes the translocation of LIPIN 1 to the nucleus. This would reduce its impact in the synthesis of triglycerides in the cytoplasm and would prepare the cell to attenuate lipid accumulation in hepatocytes through the activation of PPARα. These findings are consistent with the protective effect reported for HIF-1α induction in alcoholic fatty liver [41]. This metabolic flexibility would be lost in the presence of an excess of lipids through the reduction in succinate levels. This metabolite inhibits HIF hydroxylase enzymes and its decrease attenuates HIF-1α levels [30]. The findings of this study also suggest that challenging wild-type mice with the HFD or *Sirt3*-deficient mice with a standard diet is not enough to promote a decrease in succinate levels and that only the combination of *Sirt3* deficiency with the HFD results in the reduction of succinate. Since an excess fatty acids has been reported to reduce succinate, leading to a decrease in HIF-1α levels [30], the results of this study suggest that only the exacerbation of the accumulation of fatty acids observed in *Sirt3-*deficient mice fed the HFD leads to a decrease in hepatic succinate levels.

Additional mechanisms, including lipogenesis, may play a role aggravating hepatic steatosis in *Sirt3-*deficient mice fed the HFD compared to WT mice. *Sirt3*-deficient mice fed a standard diet have previously been reported to show an increase in the expression of one lipogenic gene only, *Scd1*, whereas the expression of other lipogenic genes and *Srebp1c* was not affected [6]. The enhanced expression of *Scd1* was attributed to the increase in hepatic saturated fatty acids [6]. In this study we report that feeding *Sirt3*-deficient mice the HFD also results in enhanced levels of the mature SREBP1 form, although the increase did not reach significance. This finding was consistent with the increase in phospho-mTOR levels in these animals, since mTORC1 regulates nuclear LIPIN 1 localization to control nuclear SREBP1c abundance [26] and also regulates the processing of SREBP1c to its mature form [27]. The slight increase in SREBP1c abundance did not significantly affect the expression of genes involved in lipogenesis. This process is therefore unlikely to be involved in the increased accumulation of triglycerides observed in *Sirt3-*deficient mice fed the HFD.

Finally, when we evaluated the expression and protein levels of genes involved in lipid uptake (VLDLR and CD36), we observed that their levels increased in the liver of *Sirt3*-deficient mice fed the HFD. The expression of these two genes is regulated by the redox transcription factor Nrf2 [34–36] and, consistent with the role of Nrf2 in the upregulation of these genes, the protein levels of its target gene NQO1 increased only in Sirt3-deficient mice fed the HFD. The increase in CD36 was confirmed in cultured human hepatoma cells, where either SIRT3 inhibition or knockdown in the presence of palmitate raised both CD36 and NQO1. However, the increase in VLDLR in these cells was observed only following SIRT3 inhibition, whereas *SIRT3* knockdown increased neither *VLDLR* mRNA nor protein levels. Several differences might account for this discrepancy (e.g. human cells compared to mice and chronic HFD compared to 24 h of palmitate incubation) but, since VLDLR is also dependent on HIF-1α [42], the increased VLDLR levels caused by Nrf2 might be affected differently by these two transcription factors in cultured cells and liver. In addition, another factor that can explain these differences is that the magnitude of the changes is likely to depend on the degree of the decrease in SIRT3 activity, being higher with the inhibitor than with *Sirt3* knockdown.

**Fig. 7:**
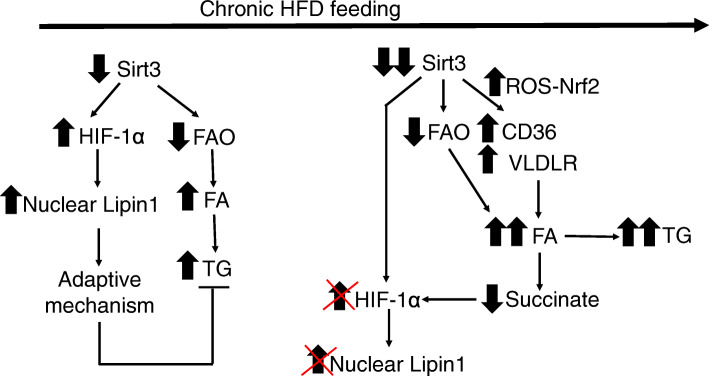
Potential new mechanisms by which Sirt3 deficiency promotes hepatic steatosis in mice fed a HFD. Exposure to a HFD reduces Sirt3 and contributes to triglyceride accumulation in the liver. However, hepatic lipid accumulation is attenuated by the activation of an adaptive mechanism involving an increase in nuclear HIF-1α and Lipin 1. Longer exposures to a HFD exacerbates Sirt3 decrease leading to higher uptake of lipids through an Nrf-2 mediated increase in CD36 and VLDLR. This results in a higher increase in fatty acid accumulation, which in turn reduces succinate levels, ultimately suppressing the adaptive increase in HIF-1α and Lipin 1. FAO: Fatty acid oxidation

Notably, chronic HFD feeding has been reported to reduce the hepatic levels of SIRT3 [6, 43]. Thus, we can envisage the following potential sequence of events in mice fed the HFD (Fig. 7). Chronic HFD feeding would reduce SIRT3 levels, which in turn would result in hyperacetylation and a subsequent decrease in the activity of enzymes involved in mitochondrial β-oxidation, thus leading to hepatic steatosis. The decrease in SIRT3 levels might activate an adaptive mechanism through the increase in nuclear HIF-1α and LIPIN 1 levels to attenuate hepatic steatosis. However, the progressive decrease in SIRT3 levels would exacerbate the increase in lipid uptake, through the upregulation of CD36 and VLDLR, respectively. Finally, the progressive accumulation of fatty acids in the cells would reduce succinate levels, thereby blunting the increase in HIF-1α and LIPIN1 and ultimately aggravating steatosis. The findings of this study performed in vitro and in vivo support the presence of a new adaptive mechanism that may offer additional strategies to treat NAFLD by regulating the HIF-1α-LIPIN 1 pathway that need to be confirmed by additional studies.

Although there is yet little evidence about whether the mechanism described in this study operates in human NAFLD, it has been reported that a unique single nucleotide polymorphism in the *SIRT*3 gene results in reduced SIRT3 enzymatic efficiency and an increased risk of developing metabolic syndrome and hepatic steatosis in humans [6]. It would be interesting to examine the translational potential of the findings of this study by conducting a study to assess whether hepatic CD36 and VLDLR are upregulated and as to whether the increase in succinate levels plays a role in accelerating the development of the disease in these patients.

## Conclusions

Overall, the findings of this study point to SIRT3 as a key protein for controlling the PPARα-target genes involved in fatty acid oxidation through the HIF-1α-LIPIN 1 pathway. The activation of this pathway is disturbed by the presence of lipids that favor the exclusion of LIPIN 1 from the nucleus, where it co-activates fatty acid synthesis. In addition, *Sirt3* deficiency promotes liver triglyceride accumulation by increasing CD36 and VLDLR levels via a Nrf2-dependent mechanism. The findings of this study open new mechanisms that can contribute to NAFLD and whose regulation may offer potential therapeutic opportunities to treat this condition.

## Supplementary information

**Additional file 1: Supplementary Figure 1.** Knockdown of SIRT3 in Huh-7 hepatocytes. mRNA (A) and protein (B) levels of SIRT3 in human Huh-7 hepatocytes transfected with control (CT) or *SIRT3* siRNA and incubated with fatty acid free-BSA or BSA-palmitate (0.5 mM) (Pal) for 24 h. a, *p* < 0.05 vs. CT siRNA cells. b, *p* < 0.05 vs. CT siRNA cells incubated with palmitate. **Table S1.** Primer sequences used for RT-PCR

## Data Availability

The datasets used and/or analysed during the current study are available from the corresponding author on reasonable request.

## Abbreviations

AAPBO: 5-amino-2-(4-aminophenyl) benzoxazole
Acox: Acyl-CoA oxidase
Cpt-1α: Carnitine palmitoyltransferase 1α
CD36: Cluster of differentiation 36
Fas: Fatty acid synthase
HFD: High-fat diet
HIF-1α: Hypoxia-inducible factor 1α
Mcad: Medium-chain acyl-CoA dehydrogenase
Keap1: Kelch-like ECH-associated protein 1
mTORC1: Mammalian target of rapamycin (mTOR) complex 1
NAFLD: Non-alcoholic fatty liver disease
NQO1: NAD(P)H:quinone oxidoreductase
Nrf2: Nuclear factor (erythroid-derived 2)-like 2
SIRT3: Sirtuin 3
PPAR: Peroxisome proliferator-activated receptor
SCD1: Stearoyl-CoA desaturase 1
SREBP: Sterol regulatory element-binding protein
VLDLR: Very low-density lipoprotein receptor
