# Supplementary material for: SIRT3 deficiency exacerbates fatty liver by attenuating the HIF1α-LIPIN 1 pathway and increasing CD36 through Nrf2
Source: Cell Commun Signal. 2020 Sep 10;18:147. doi: 10.1186/s12964-020-00640-8 (PMC7488148; doi:10.1186/s12964-020-00640-8)
Supplement: Supplementary file 2 — Additional file 1: Supplementary Figure 1. Knockdown of SIRT3 in Huh-7 hepatocytes. mRNA (A) and protein (B) levels of SIRT3 in human Huh-7 hepatocytes transfected with control (CT) or SIRT3 siRNA and incubated with fatty acid free-BSA or BSA-palmitate (0.5 mM) (Pal) for 24 h. a, p < 0.05 vs. CT siRNA cells. b, p < 0.05 vs. CT siRNA cells incubated with palmitate. Table S1. Primer sequences used for RT-PCR [file 12964_2020_640_MOESM2_ESM.pdf]

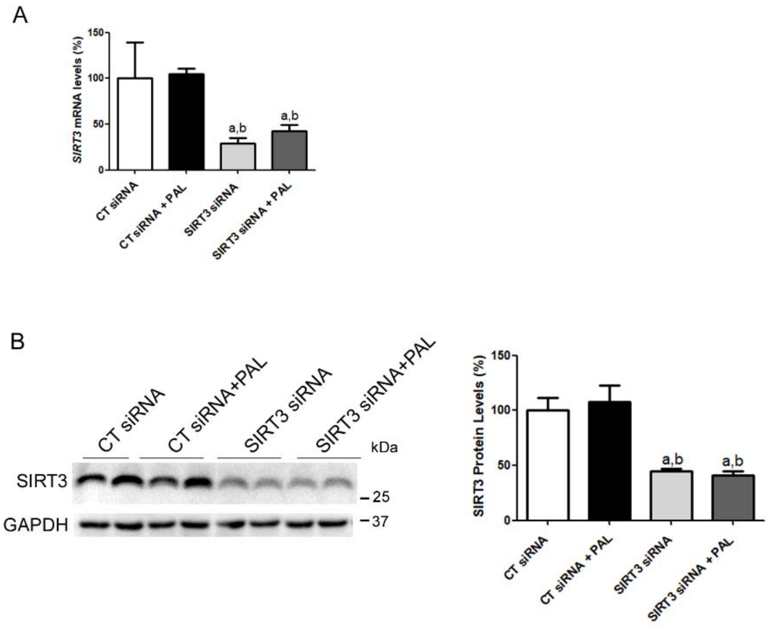

Supplementary Figure 1. Knockdown of SIRT3 in Huh-7 hepatocytes.

mRNA (A) and protein (B) levels of SIRT3 in human Huh-7 hepatocytes transfected with control (CT) or *SIRT3* siRNA and incubated with fatty acid free-BSA or BSA-palmitate (0.5mM) (Pal) for 24 h. a, $p<0.05$ *vs.* CT siRNA cells. b, $p<0.05$ *vs.* CT siRNA cells incubated with palmitate.

Table S1. Primer sequences used for RT-PCR

Gene	Primers	
mAprt	for	5’-CAGCGGCAAGATCGACTACA-3’
	rev	5’-AGCTAGGGAAGGGCCAAACA-3’
mAcox	for	5’-TCTGGAGATCACGGGCACTT-3’
	rev	5’-TTTCCAAGCCTCGAAGATGAG-3’
hCPT1α	for	5’-TGCTTTACAGGCGCAAACTG-3’
	rev	5’-TGGAATCGTGGATCCCAAA-3’
mCpt1α	for	5’-GCAGAGCACGGCAAAATGA-3’
	rev	5’-GGCTTTCGACCCGAGAAGAC-3’
mFas	for	5’-CATTGGTGGTGTGGACATGGT -3’
	rev	5’-GACCGCTTGGGTAATCCATAGA-3’
mFgf21	for	5’-CAGGGAGGATGGAACAGTGGTA-3’
	rev	5’-TGACACCCAGGATTTGAATGAC-3’
hGAPDH	for	5’-GGCCTCCAAGGAGTAAGACC-3’
	rev	5’-AGGGGTCTACATGGCAACTG-3’
mMcad	for	5’-TGACGGAGCAGCCAATGA-3’
	rev	5’-ATGGCCGCCACATCAGA-3’
mPparα	for	5’-CAAGGCCTCAGGGTACCACTAC-3’
	rev	5’-GCCGAATAGTTCGCCGAAA-3’
mScd1	for	5’-CTGTACGGGATCATACTGGTTC-3’
	rev	5’-GCCGTGCCTTGTAAGTTCTG-3’
hSIRT3	for	5’-CGGCTCTACACGCAGAACATC-3’
	rev	5’-AGGTTCCATGAGCTTCAACCA-3’
hVLDLR	for	5’-CAAGAGGAAGTTCCTGTTTAACTCTGA-3’
	rev	5’-TGACCAGTAAACAAAGCCAGACA-3’
mVldlr	for	5’-TCCAATGGCCTAATGGAATTACA-3’
	rev	5’-AGCATGTGCAACTTGGAATCC-3’
